# Management of a Serratia marcescens outbreak in a neonatal unit – improving hand hygiene does the job

**DOI:** 10.1186/2047-2994-4-S1-P224

**Published:** 2015-06-16

**Authors:** I Soulake, A Gayet-Ageron, DG Pichoud, R Pfister, G Renzi, D Pittet, W Zingg

**Affiliations:** 1 Infection Control Programme, University of Geneva Hospitals (HUG), Geneva, Switzerland; 2 Paediatrics, University of Geneva Hospitals (HUG), Geneva, Switzerland; 3 Microbiologic Laboratory, University of Geneva Hospitals (HUG), Geneva, Switzerland

## Introduction

Many outbreaks due to *Serratia marcescens* among neonates have been described in the literature. While the source was rarely identified, the emphasis primarily was given to the role of the environment on the chain of transmission.

## Objectives

To describe a *S. marcescens* outbreak and to evaluate the impact of the control measures implemented.

## Methods

Between February and March 2013, 2 infants and 2 neonates were found to be colonized by *S. marcescens* in our tertiary care university-affiliated hospital. The two infants were hospitalized in the neonatal unit before having been transferred to the unit of small infants. An investigation was launched with environmental sampling in April 2013 and five point prevalence surveys (nasopharyngeal and rectal swabs) were performed between mid-April and mid-June. All identified pathogens were genotyped. Audits of best practices and hand hygiene (439 direct observations) were performed and an intensive hand hygiene promotion programme offered.

## Results

A total of 160 environmental samples were obtained and 202 neonates were screened. Twenty-three neonates were found to be colonized by *S. marcescens*, which were all genotypically identical (attack rate = 11.39%). No invasive infections due to *S. marcescens* occurred. Hand hygiene compliance improved from 51% in April 2013 to 79% in May 2013 following the training programme, and remained high in the following months. No formal source was identified and all environmental samples were negative. No *S. marcescens* were identified in point prevalence surveys conducted in June and October 2013.

## Conclusion

Improving best practices and particularly hand hygiene proved to be effective in ending this outbreak, which highlights the role of hand hygiene in the successful management of *S. marcescens* outbreaks among neonates.

## Disclosure of interest

None declared.

